# Prevalence and Associated Risk Factors of *Salmonella*, *Shigella*, and Intestinal Parasites among Food Handlers in Motta Town, North West Ethiopia

**DOI:** 10.1155/2020/6425946

**Published:** 2020-01-13

**Authors:** Tibebu Yesigat, Mohabaw Jemal, Wubet Birhan

**Affiliations:** ^1^Hulet Ejju Enessie Woreda Health Office, Motta, Ethiopia; ^2^University of Gondar, College of Medicine and Health Sciences, School of Biomedical and Laboratory Sciences, Department of Medical Microbiology, Gondar, Ethiopia

## Abstract

**Background:**

Intestinal parasite, *Salmonella*, and *Shigella* infections are the main public health concerns in the world, especially in developing countries due to inaccessibility of safe water and unhygienic food handling practices of food handlers.

**Methods:**

A cross-sectional study was conducted in food handlers in Motta town, from February 2019 to April 2019. The study was undertaken to determine the prevalence and associated risk factors of *Salmonella, Shigella*, and intestinal parasites among 243 food handlers. Sociodemographic and risk factors were collected using pretested structured questionnaire. The stool sample was collected and examined with direct wet mount and concentration techniques. Culture was performed using xylose lysine deoxycholate agar and MacConkey agar and biochemical tests like Klinger iron agar (KIA), lysine iron agar (LIA), Simmons citrate agar, sulphide indole motility test, citrate utilization, and urease production test were conducted to isolate *Salmonella* species and *Shigella* species. An antibiotics susceptibility test was performed with Mueller-Hinton agar using the disk diffusion method. Data were entered using statistical package Epi-Data Version 3.1 and analysed with logistic regression using SPSS version 25 and Fisher's exact test. A *p value* < 0.05 at 95% CI was considered as statistically significant.

**Results:**

The prevalence of intestinal parasite, *Salmonella*, and *Shigella* was 27.6%, 2.5%, and 1.6%, respectively, and hookworm was the predominant intestinal parasite detected in the stool. Antimicrobial resistance was observed in ampicillin and tetracycline (100%) in *Salmonella* species and *Shigella* species. Risk factors like fingernail status, fruit washing before eating, cleaning utensils, and regular shoe wearing habit were associated with intestinal parasite, whereas fingernail status and wearing kitchen gown during food service were significantly associated with *Salmonella* and *Shigella* infections.

**Conclusion:**

The prevalence of intestinal parasitic infections, *Salmonella*, and *Shigella* infections in this study indicates the importance of food handlers as probable sources of enteropathogenic infections. Food handlers should have follow-up on the order of food safety rules and keep their personal hygiene. Hotel owners are responsible to control the health status and their created awareness by given food hygiene training for food handlers. Therefore, policy-makers and implementers should focus on the risk factors to reduce the prevalence below the level of public health importance.

## 1. Introduction

Food borne diseases (FBD) are an important cause of morbidity and mortality in the world. It causes the highest challenges in developing and developed countries [1, 2]. There are different challenges in identifying the burden of FBD in low- and middle-income countries (LMICs) due to unavailability of data [3]. Based on the WHO annual report, about 30% of world population is affected by food-borne disease and nearly about 2 million mortality rates and 70% of the diarrheal disease cases are associated with the consumption of contaminated food in developing countries [4, 5]. In contrast to these reports, up to 10% population of industrialized countries is affected by FBD [6]. Food-borne disease outbreaks in the United States with unsafe food handling practice in institutional establishments and homes were 79% and 20%, respectively [7]. Most of these infectious diseases are caused by enteropathogenic bacteria, including *Salmonella* species, *Shigella* species, and intestinal parasites (IP) which include helminths and protozoan infections [8]. The major IP that cause infections are *Tania saginate, Hymenolepis nana, Ascaris lumbricoides, Strongyloides stercoralis, Trichuris trichiura, Enterobius vermicularis*, and hookworms, and the most prevalent intestinal protozoan diseases reported are *Giardia lamblia* and *Entamoeba histolytica* [9].

Intestinal parasitic infections (IPIs), caused by either intestinal protozoan or helminths or both, are the main public health concern in the world, especially in LMICs due to inabilities in securing safe water and food handling practices [10]. These regions of developing countries are characterized by low levels of education, hygiene, and sanitation practice and a high disease burden of intestinal parasitic infections [11].

Food handlers with poor personal hygiene, working in the food service establishments, can be infected by different enteropathogens, possibly causing fecal contamination of foods by their hands during food preparation, which may be the cause for the transmission of infections to the local community [12]. Medically important IP are transmitted through ingestion of food or water contaminated with viable stages of these parasites [13, 14]. Finally, there may be the transmission of these enteropathogenic infections to the public in the local community [15].

Therefore, there is a need of periodic assessments of IP prevalence for the intervention of high-risk groups and food handlers to combat food-borne diseases. As a result of this, the Federal Ministry of Health (FMoH) of Ethiopia has prioritized intestinal helminth infection as one of Neglected Tropical Diseases (NTDs) in the National Master Plan of NTDs, to address public health problems. Most of the WHO lists of NTDs in Ethiopia are soil-transmitted helminth parasites (*A. lumbricoides*, *T. trichiura*, hookworm, and schistosomiasis) [16].

Genus *Salmonella* is a member of Enterobacteria, Gram-negative, nonlactose fermenter, motile and gas producer, and flagellated facultative anaerobic rod bacilli [17]. *Salmonella* invades the mucosa of the small and large intestine that causes an inflammation, and invasion of epithelial cells induces an inflammatory reaction which causes diarrhea [18]. *Salmonella* infection is most commonly occurring in countries with poor standards of hygiene in food preparation, handling, and sewage disposal system. The carrier states of food handlers are of concern of food service establishments because of the risk of contamination of food [19]. Food-borne salmonellosis is often transmitted by eating unwashed fresh fruits and vegetables and contaminated food, and not thoroughly cleaning work surfaces used to prepare raw meat and other foods in the kitchen is the source of *Salmonella* infection [20]. The problem is more severe in developing countries like Ethiopia where there is a lack of personal hygiene and food safety measures and empirical treatment is practiced. Drug resistance in *Salmonella* is quick because *Salmonella* species enables to exchange and keep different genes responsible for antimicrobial resistance. Each day, new mutations in genes responsible for antimicrobial resistance are happening [21].

*Shigella* is the member of Enterobacteriaceae, nonlactose fermenter, nonmotile, and nongas producing Gram-negative rods. There are four species of *Shigell*a, which include *S. dysenteriae (group A), S. flexneri (group B), S. boydii (group C)*, and *S. sonnei (group D)* which cause the disease shigellosis. Shigellosis is an acute invasive enteric bacterial infection, which is clinically manifested by frequent bloody diarrhea [22]. Shigellosis is common in many developing countries, and it occurs in epidemics causing considerable morbidity and mortality. Annually, there are 165 million cases of shigellosis resulting in 1.1 million deaths in the developing world [23]. The possible associated risks of shigellosis are bare hand contact with the food followed by failure to properly wash hands, inadequate cleaning of processing or preparation equipment or utensils, and cross-contamination of ready-to-eat foods with contaminated raw ingredients [24].

*Shigella* is the emerging drug-resistant species that was indicated in a few research studies conducted in the country [25, 26]. *Salmonella* and *Shigella* are among the common bacterial pathogenic organisms with high multidrug-resistance (MDR) patterns globally, resistant to at least one antibiotic for three or more antimicrobial classes of drugs which is increasing from time to time in developing countries [27–29]. This study aims to determine the prevalence, antimicrobial susceptibility patterns, and associated risk factors of *Shigella, Salmonella*, and intestinal parasites among food handlers in Motta town, North West Ethiopia.

## 2. Materials and Methods

### 2.1. Study Design and Area

An institution-based cross-sectional study was conducted from February 2019 to April 2019 in the town. The town is located 365 km from the capital city of the country, Addis Ababa, 120 km far from Bahir Dar, which is the capital city of the Amhara regional state and 196 km away from Debre Markos, the capital city of East Gojjam zone. The town had a total population of 44,914 (19,972 males and 24,942 females) [30]. In Motta town, there are 10 hotels, 26 restaurants, and 47 cafeterias with a total of 255 (47 males and 208 females) food handlers.

### 2.2. Study Population and Sample Size

The study population was food handlers working in hotels, restaurants, and cafeterias in Motta town within the study period. All food handlers working in hotels, restaurants, and cafeterias were included in the study because of the small number of study population.

### 2.3. Data Collection Tools and Procedure

#### 2.3.1. Sociodemographic and Personal Hygiene Practice Data Collection

Data related to sociodemographic factors, food handling practices, and other related factors were collected using a pretested questionnaire. Data on fingernail trimming status and cleanness of toilet facility were observed and recorded.

#### 2.3.2. Stool Specimen Collection and Microscopic Examination

Collect a small amount of fresh stool sample (3–5 g or 4 ml if diarrhoea) with a clean and tight-lid sample container. Parasitological microscopic stool examination was immediately performed by emulsifying uniformly about a matchstick size of stool (2 mg) in a drop of normal saline (0.85% NaCl). An iodine wet mount was mainly used to stain the nucleus of the protozoa cysts. The sedimentation technique was performed using the readily modified formalin-ether sedimentation technique [31, 32]. Stool samples collected for *Salmonella* and *Shigella* culture were refrigerated at 4°C with the Cary-Blair transport medium and transported to the Amhara Public Health Institution microbiology laboratory within 24 hrs of collection.

#### 2.3.3. Culture and Isolation of *Salmonella* and *Shigella*

Stool samples preserved in the Cary-Blair transport media were transferred to Selenite F broth (Oxoid) and incubated for 18 h at 37°C. Following the incubation of Selenite F broth, a loop full of samples was streaked to XLD (Oxoid) and MacConkey agar (MAC) (Oxoid) was incubated at 37°C for 24 h. The growth of *Salmonella* species and *Shigella* species were detected by their characteristic appearance on XLD agar (*Shigella*: red/pink colonies, *Salmonella*: red with some times black centre [33, 34]. For confirmation, at least 1–3 presumptive colonies were selected and purified by streaking on to MAC (Oxoid) plates and incubated at 37°C for 24 h. Pure colonies with white/colourless colonies that grow on MAC were used for biochemical tests. The biochemical tests used for final identification were Klinger iron agar (KIA), lysine iron agar (LIA), Simmons citrate agar, sulphide indole motility test (motility, H_2_S production, indole), citrate utilization, and urease production test [33, 34].

#### 2.3.4. Antimicrobial Susceptibility Testing

Antimicrobial susceptibility testing was conducted by the Kirby-Bauer disk diffusion technique according to the criteria set by the Clinical Laboratory Standard Institute (CLSI) 2019 guideline [35]. The inoculums were prepared by picking 3–5 pure colonies of similar test organisms with a sterile wire loop and were suspended in sterile normal saline. Density of the suspension inoculated was determined by comparison with opacity standard on McFarland 0.5 barium sulphate solution. The test organisms were uniformly seeded over the Mueller-Hinton agar (Oxoid, Basingstoke, Hamsphire, England) surface. A sterile cotton swab was dipped into the adjusted suspension, and excess was removed by gentle rotation of the swab against the inside wall of the tube. The swab was inoculated evenly over the entire surface of Mueller-Hinton agar (Oxoid), and then the inoculated plates were allowed to air dry for 15 minutes. The antibiotic disks were placed aseptically on the plate using sterile forceps, and plates were incubated at 37°C for 24 hours [35].

All *Salmonella* species and *Shigella* species isolates were tested against the antimicrobial disks amoxicillin-clavulanate (30 *μ*g), chloramphenicol (30 *μ*g), ampicillin (10 *μ*g), trimethoprim-sulfamethoxazole (1.25/23.7 *μ*g), ciprofloxacin (5 *μ*g), tetracycline (30 *μ*g), and cefotaxime (30 *μ*g) [35]. Antimicrobial agents were selected based on the criteria of Clinical and Laboratory Standard Institute (CLSI) 2019 guideline. Finally, the diameters of the zone of inhibition around the discs were measured to the nearest millimetres using a ruler and classified as sensitive, intermediate, and resistant according to the standardized table supplied by CLSI 2019 guideline clinical breakpoints [35].

### 2.4. Data Quality Control

The data collector was trained in the methods of data collection technique. Completeness and clarity of the collected data were checked every day. A pretested, structured questionnaire was used for data collection on sociodemographic characteristics and associated risk factors. The questionnaire was initially prepared in English and translated into the local language, Amharic.

### 2.5. Laboratory Quality Control

The sterility of culture media was checked by incubating about 5% batch of the media at 35–37°C overnight and evaluated for possible contamination. Standard reference strains of *S. typhimurum* (ATCC-14028) and *E. coli* (ATCC-25922) were used as a quality control throughout the study for culture [35]. Data quality was ensured at various activities of the study by following the prepared standard operating procedure (SOP) of the laboratory.

### 2.6. Data Analysis and Interpretation

Data were entered and coded with statistical package Epi-Data version 3.1 and analysed using SPSS version 25 software. Descriptive statistics were used to summarize sociodemographic and prevalence of enteropathogens. Bivariate and multivariate logistic regression analyses were carried out to identify potential factors for the prevalence of *Salmonella, Shigella*, and intestinal parasite infections. Adjusted odds ratio (AOR) at 95% confidence intervals (CI) was used to measure the association between potential risk factors and enteric pathogen prevalence. Those variables at a cut-off point *p* value < 0.2 in bivariate analysis were candidate for multivariate analysis. Fisher's exact test was used for analysis of *Salmonella* and *Shigella* possible associated factors. A *p* value < 0.05 at 95% CI was considered as statistically significant.

### 2.7. Ethical Considerations

Ethical clearance was obtained from School of Biomedical and Laboratory Sciences, University of Gondar ethical review committee, and a letter informing the Motta town health office about the purpose of the study was written by School of Biomedical and Laboratory Sciences, University of Gondar. Support letters were obtained from the Motta town health office and permissions from the food establishment administration prior to data collections. Consent was obtained from study participants after explaining the purpose and objective of the study. Study participants who were not willing to participate in the study were not forced to participate. Study participants were informed that all data and sample obtained from them were kept confidential by using codes instead of any personal identifiers and is meant only for the purpose of the study. For those study participants who were positive, we advised to go to the health institution to consult the clinicians for medical treatment and treated with the respective antibiotics and antiparasitic drugs. The treatment cost of those positive study participants was covered by the food establishment institution and woreda health office, specifically the health center.

## 3. Results and Discussion

### 3.1. Results

#### 3.1.1. Sociodemographic Characteristics

A total of 243 food handlers were enrolled in this study, of whom 201 (82.7%) were female. The median age was 22 with an age range of 13 to 50 years. One hundred and thirty-three (54.7%) of the food handlers were with service years of below one year. Almost all (97.9%) of the food handlers were not certified in food handling training. About 82 (33.7%) of the food handlers were of a primary school (grade 5–grade 8).

All food handlers did not have a regular medical check-up, and they transferred food with bare hands during meal serving (Table 1).

#### 3.1.2. Prevalence and Associated Risk Factors of Intestinal Parasites

Six parasite species were identified from food handlers with an overall prevalence of 27.6% (67/243) (95% CI: 21.5–32.9). The predominant parasitic infections identified were hookworm 37.3% (25/67) followed by *E. histolytica/dispar* 29.9% (20/67), *G. lamblia* 17.9% (12/67), *A. lumbricoid* 8.9% (6/67), Tania spp. 6% (4/67), and *S. Stercolaris* 4.5% (3/67). The prevalence of parasitic coinfections in food handlers were 4.5% (3/67), of which *E*. *histolytica/dispar* and hookworm were observed in two study participants and one of the study participants was coinfected with hookworm and Tania spp. These parasitic infections were identified from food handlers who did not have regular medical check-up. The prevalence of parasitic infections was high on food handlers that did not properly wash utensils 58.1% (48/74), and food handlers with untrimmed fingernail status had the prevalence of 45.6% (47/103). The parasitic infections were also high in food handlers who did not regularly wash their hands with soap after toilet, 43% (43/100), and those that did not wash fruits before eating were 44.7% (51/114).

From the variables which were computed in bivariate logistic regression analysis, 10 variables met a *p* value <0.2. All those variables that were associated with IPIs at a *p* value <0.2 in bivariate logistic regression analysis were computed in a multivariate logistic regression analysis. Multivariate analysis of study subjects showed that food handlers with untrimmed finger nail status were 4.067 times more likely to have increased parasitic infections when compared with food handlers with trimmed fingernail status (AOR = 4.067; 95% CI, 1.832–9.027, *p*=0.001). In addition, food handlers who did not regularly wear shoes were 2.311 times more likely to have increased IPIs as compared with respondents who wore shoes regularly (AOR = 2.311; 95% CI, 1.020–5.233, *p*=0.045). Food handlers who did not wash fruits before eating were 4.428 times at risk to IPIs (AOR = 4.428; 95% CI: 1.857–10.557, *p*=0.001), and study participants that did not properly clean utensils were 5.690 times at risk for IPIs (AOR = 5.690, CI: 2.382–13.595, *p* < 0.001) (Table 1).

#### 3.1.3. Prevalence and Associated Risk Factors of *Salmonella* and *Shigella* Isolates

The overall prevalence of *Salmonella* or *Shigella* among food handlers was 4.1% (10/243) (95% CI: 2.1–6.6). Out of the 243 food handlers screened, *Salmonella* was 2.5% (6/243) (95% CI: 0.8–4.5) and *Shigella* was 1.6% (4/243) (95% CI: 0–3.2). All bacterial isolates were identified from food handlers who did not have regular medical check-up, food hygiene training, and touching food with bare hands. Regarding educational level, the highest (20%) prevalence of *Salmonella* and *Shigella* was observed among study participants who only read and write. Those food handlers with untrimmed fingernail status had 7.8% (8/103) bacteria prevalence. These bacterial infections were high in food handlers who did not regularly wash their hands with soap after toilet 7% (7/100) and did not wear kitchen coat during food preparation 7.2% (8/111). About 3 (50%) of *Salmonella* carrier food handlers had been coinfected with intestinal parasites of hookworm, *G. lamblia*, and E. *histolytica/dispar* of each, whereas one study participant (25%), *Shigella* carrier, was coinfected with *G. lamblia*. From the variables which were analyzed using Fisher's exact test, two variables (fingernail trimming status and wearing kitchen coat during food preparation) were significantly associated with bacterial infection (*pvalue* < 0.05). Food handlers with untrimmed fingernail status were 5.811 times more likely to be positive for *Salmonella* or *Shigella* compared with those who trimmed their finger nails (OR = 5.811, 95% CI = 1.207–27.967, *p-value* = 0.020). Similarly, the odds of being positive for *Salmonella* or *Shigella* were 5.049 times higher among food handlers who did not wear a kitchen gown during food preparation, compared with those who wore a kitchen coat (OR = 5.049, 95% CI = 1.049–24.289, *pvalue* = 0.047). But, there was no association among other characteristics like washing hands with soap after toilet, before eating food, and before preparing food, use of common knife, cleaning utensils, and availability of clean toilet (*pvalue* > 0.05) (Table 2).

#### 3.1.4. Antimicrobial Susceptibility Patterns of *Salmonella* and *Shigella* Isolates

In the current study, six [6] *Salmonella* and four [4] *Shigella* isolates were tested against seven antimicrobial agents (Table 3). The results obtained showed that the organisms have variations in their susceptibility pattern to all the antimicrobials used.

The highest degree of resistance detected among the seven antimicrobials for *Salmonella* was for ampicillin (6/6) followed by tetracycline (5/6) resistance and (1/6) intermediate resistance. It was also resistant to amoxicillin-clavulanate (3/6), for trimethoprim-sulfamethoxazole, and cefotaxime (1/6). *Salmonella* was (6/6) susceptible to chloramphenicol and ciprofloxacin. All ampicillin (6/6)-resistant *Salmonella Spp.* isolates were resistant to tetracycline (6/6) and susceptible to chloramphenicol (6/6) and ciprofloxacin (6/6) and trimethoprim-sulfamethoxazole and cefotaxime (5/6).

*Shigella* isolates were 100% (4/4) resistant to ampicillin and tetracycline and 75% (3/4) resistant to amoxicillin-clavulanate . It was also 25% (1/4) resistant to chloramphenicol and 25% (1/4) resistant and 25% (1/4) intermediate resistant to cefotaxime.

*Shigella* was 100% (4/4) susceptible to trimethoprim-sulfamethoxazole and ciprofloxacin. All ampicillin (4/4)-resistant *Shigella* strains were resistant to tetracycline, and all *shigella* isolates (4/4) were susceptible to trimethoprim–sulfamethoxazole and ciprofloxacin. No *Shigella* and *Salmonella* isolates were susceptible to all the seven tested antibiotics, and as well, no *Shigella* and *Salmonella* isolates were resistant to all the seven antibiotics tested (Table 3).

**Figure 1:**
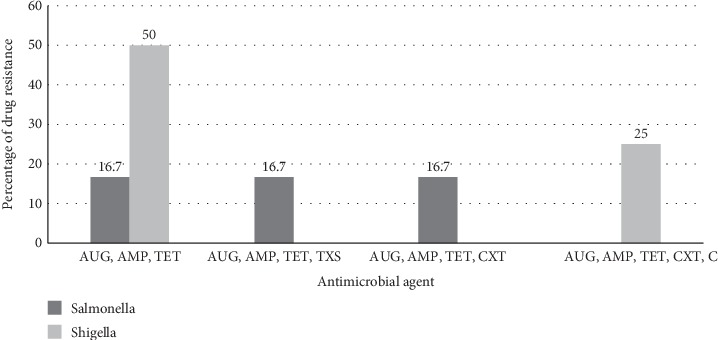
Multidrug resistance pattern of *Salmonella* and *Shigella* among food handlers in Motta town. AUG, amoxicillin-clavulanate, TET, tetracycline, AMP, ampicillin, TXS, trimethoprime-sulfamethoxazole, CXT, cefotaxime, and C, chloramphenicol.

Multidrug resistance was detected in 50% (3/6) of *Salmonella* isolates and 75% (3/4) of *Shigella* isolates resistant to at least one antibiotic for three or more antimicrobial classes of drugs (Figure 1).

## 4. Discussion

Food handlers of catering establishments might be carrying a wide range of enteropathogens, which implicates in the transmission of many infections to the public in the community and in university students. The spread of disease via food handlers is a common and persistent problem worldwide [36]. The parasitic infection prevalence 27.6% (95% CI: 21.5–32.9) lies between the low carriage rate of 3.7% and the high rate of 83.3% in different areas of the world [14,37]. This prevalence of parasitic infection among food handlers was in agreement with the findings of other studies conducted in Ethiopia like Gondar town 29.1% [38] and 25% [39], Haramaya 25.2% [40], in Khartoum state, Sudan 30.5% (64), 23.7% [41] in Kenya, and Eldoret town 30.4% [11]. The higher prevalence rate of parasites was reported in Ethiopia from Bahir Dar 41.1% [42], Addis Ababa 45.3% [36], Mekelle 52.4% [4], Arba Minch 36% [43], Wolita Sodo 41% [28] and 33.7% [44], Yebu town 44.1% [13], Gambia 46.3% [45], and Swat Pakistan 83.3% [14]when compared with the present study. However, lower prevalence was reported in Axum 14.5% [46], Bahir Dar 12.9% [47], Kenya Nairobi 15.7% [48], Sari Iran 15.5% [15], Western Iran 9% [49], and Northern Iran 3.7% [36]. This discrepancy might be due to epidemiologic distribution difference, personal hygiene practices and environmental sanitation difference, proper cleaning of utensils, fruit washing, and regular medical check-up difference.

In this study, from 67 total positive samples, the predominant parasite from food handlers was hookworm 25 (37.3%). This was similar to the study conducted in Bahir Dar town which identified hookworm as the commonest parasite 26(48.2%) [47]. The second and third prevalent IPIs identified were *E. histolytica/dispar* and *G. lamblia,* which were 29.9% and 17.9%, respectively. These protozoa account for about 47.8% of IPIs in this study. Thus, food handlers harbouring these protozoan parasites that do not need environmental maturation might contaminate food and water and spread the parasites directly to customers in food establishments. High prevalence of hookworm is a good indicator of improper faecal disposal, untrimmed fingernail status, and not regularly wearing shoe, whereas that of ameobiasis and giardiasis reflects improper water and food handling practice and low personal hygiene among study participants [44].

Poor practice of cleaning utensils, untrimmed fingernail status, eating unwashed fruits, and no regular wearing of shoes were identified as significantly associated risk factors of food handlers being infected by IPs. Likewise, higher prevalence of these parasites in untrimmed fingernail food handlers was reported from a study conducted in Ethiopia, Arba Minch [43], Yebu town [13], and Haramaya University [40] which results in the contamination of food and drinks during meal serving and preparing food.

In the present study, 4.1% of food handlers had either *Salmonella* or *Shigella* spp., indicating that food handlers in mass catering establishments are potential sources of salmonellosis and shigellosis infection in the community. The prevalence of *Salmonella* (2.5%) (95% CI: 0.8–4.5) among food handlers was in agreement with the studies conducted in Ethiopia like Bahir Dar 2.7% [50] and 1.6% [42] and Addis Ababa 3.5% [36]. Higher prevalence of *Salmonella* was reported in Ethiopia, Arba Minch University 6.9% [25], WolitaSodo 8.8% [28], Nigeria 17% [51], Pakistan 9.1% [52], and Jordan 6.3% [29]. However, no *Salmonella* isolate was reported in Gondar [38], Hawassa [24], and Jordan [53]. The discrepancy of these bacterial isolates in different areas may be due to the difference in the pathogen isolation technique, personal hygiene, level of environmental sanitation, epidemiologic distribution of pathogens, and sample size. For example, in Jordan, they used only XLD culture media and did not use any enrichment media.

About 1.6% (95% CI: 0–3.2) of the study participants were carriers of *Shigella* isolates, which lies between the low carrier rate of 0% and the high rate of 3.1% in different areas of the world [38,53]. The prevalence rate was comparable with studies conducted in Bahir Dar 1.2% [40], Gondar 3.1% [38], Hawassa 0.4% [24], Arba Minch university 3% [25], and Jordan 1.4% [29]. But, no *Shigella* isolate was identified in studies conducted in Addis Ababa [36] and Jordan [53]. This difference might be due to the culture and the transport media used and the geographic distribution of the bacteria. For example, in Addis Ababa, during sample collection, they did not use transport media, and this might compromise the isolation of *Shigella* species.

Prevalence of *Salmonella* and *Shigella* was significantly associated with untrimmed fingernail status. The associations in untrimmed fingernail food handlers were consistent with studies conducted at Arba Minch [25]. Untrimmed fingernail might harbour these bacteria isolates and could serve as a vehicle for transport of organisms from the source to the food due to the area beneath a fingernail harbors most organisms and is difficult to clean it. We also found a significant association of *Salmonella* and *Shigella* among food handlers who did not wear a kitchen gown during food preparation. The reason might be due to the cross infections of *Salmonella* and *Shigella* by the hands which were contacted with unwashed and pathogen-contaminated dressings to other food items during food servings that might cause for the infection of food handlers and consumers. This finding indicates that food handlers who carry these bacteria might play a role in the food-borne spread and sudden outbreaks of *Salmonella* or *Shigella* food poisoning in food handlers at large in the community. It is known that *Shigella* and *Salmonella serotype typhi* (*Salmonella typhi*) do not have a natural reservoir except human beings [47]; therefore, the primary modes of transmission are associated with poor sanitation and hygiene practices of food handlers. Hence, food handlers who carry *Salmonella* or *Shigella* should not be allowed to work in food and drinking establishments until they are treated and cured.

Antimicrobial resistance has been recognized as an emerging worldwide problem both in developed and developing countries [54]. In this study, all (100%) of the *Salmonella* isolates tested were resistant to ampicillin and tetracycline. This finding was in line with the studies conducted in Bahir Dar [42] and Addis Ababa [36] with 100% resistance to ampicillin and Nigeria with100% resistance to ampicillin and tetracycline [51]. The high proportion of resistance found in ampicillin and tetracycline could be due to the fact that it has broad antimicrobial coverage, the less expensive orally administered antibiotic and is readily available over the counter in many settings. About 50% of *Salmonella* isolates were resistant to amoxicillin-clavulanate. This was in agreement with the study conducted in Nigeria [51]. But, trimethoprim-sulphamethoxazole resistance (16.7%) disagree with the study conducted in Bahir Dar (83.3%) [42], and no resistance of *Salmonella* to chloramphenicol in our study had a discrepancy with the study conducted in Bahir Dar 33% [42] and Nigeria 59% [51]. These might be due to the potency of the used drugs prescribed in the community, distribution of drug resistance bacteria, and unrestricted use of antimicrobials in the community. *Salmonella* was (100%) susceptible to ciprofloxacin is in line with studies conducted in Addis Ababa 100% [36] and Jimma 100% [27] which might be due to the recently available drugs in the country.

*Shigella* was 100% resistant to ampicillin and tetracycline, which were similar to the study conducted in Jimma [27] and Gondar [50] with100% resistance to ampicillin. It could be due to broad antimicrobial coverage and the least expensive orally administered antibiotics and unrestricted use of the drugs in the community. It was (100%) susceptible to ciprofloxacin, which is comparable with studies conducted in Gondar 100% [54] and Jimma 100% [27]. The susceptibility of *Shigella* for trimethoprim-sulfamethoxazole (100%) was in agreement with the study in conducted in Arba Minch (100%) susceptible [25]. But, the resistance of *Shigella* to trimethoprim-sulfamethoxazole (58.8%) in Gondar [54] has discrepancy with our study (100%) which may be due to the potency of the drugs and drug resistance strain prevalence in the community.

The low resistant rate of *Salmonella* and *Shigella* isolates to chloramphenicol, 0% and 25%, respectively, could be the reason why physicians stopped to prescribe the drug long time ago and once again the strains started to become sensitive [27]. When the results of antimicrobial susceptibility patterns of *Shigella* and S*almonella* isolates in this study were compared with earlier reports of Ethiopia, it showed that there has been a change in the susceptibility patterns of antibiotic use. It showed a high degree of resistance to the commonly used antimicrobials. According to this study, ampicillin, tetracycline, and amoxicillin-clavulanate are no longer effective for the treatment of shigellosis and salmonellosis in the study area. About 75% of *Shigella* and 50% of S*almonella* isolates of the study are multidrug resistant. Higher MDR isolates observed in this study might be due to administration of multiple antimicrobials for infections and indiscriminate use of antibiotics. About 94.1% of *Shigella* isolates and 100% of *Salmonella* isolates were multidrug resistant in a study conducted in Gondar which was higher than in our study [50]. A study conducted in Addis Ababa showed that all *Salmonella* isolates were multidrug resistant. In addition to ciprofloxacin, both *Shigella* and *Salmonella* were 100% sensitive to trimethoprim-sulfamethoxazole and chloramphenicol, respectively. Therefore, physicians and clinicians should use the respective drugs as the first-line antibiotics for the treatment of shigellosis and salmonellosis in the study area.

## 5. Conclusion and Recommendations

The total rate of isolated pathogens from stool was 27.6% intestinal pathogenic parasite, 2.5% *Salmonella*, and 1.6% *Shigella*. The majority of risk factors were significantly associated with IPIs and *Salmonella* and *Shigella* infections. Therefore, measures including regular medical check-up, improved safe food handling practice, improved personal hygiene and environmental sanitation, and improved facility utensils sanitation should be considered in catering establishments. Antibiotics like ampicillin, tetracycline, and amoxicillin-clavulanate developed resistance to *Shigella* and *Salmonella* species. About 50% of *Salmonella* and 75% of *Shigella* isolates develop multidrug resistance to the commonly used antibiotics. Revising the national treatment guideline on commonly used antibiotics can significantly reduce the development of MDR of Salmonella and Shigella.

To control an increased prevalence of parasitic infection and bacterial infection, there should be regular parasitological, microbiological, and antimicrobial surveillance. Food handlers should have regular medical check-up and food hygiene training. Awareness should be created for food handlers about risk factors like washing of fruits and vegetables, proper cleaning of utensils, trimming of fingernail status, wearing a kitchen gown during food service, and regular shoe wearing to prevent enteropathogenic infections. Health education or training should be given to food handlers about personal hygiene, environmental sanitation, and food handling practice. Further research is recommended to validate the source and point of enteropathogenic infection as well as molecular characterization, and serotyping of *Salmonella* and *Shigella* is important.

## Figures and Tables

**Table 1:** Bivariate and multivariate analyses of risk factors associated with intestinal parasites in food handlers in Motta town, North West Ethiopia.

Variable (*n* = 10)	Neg. *N* (%)	Pos. *N* (%)	Total *N* (%)	COR (95% CI)	*p* value	AOR (95% CI)	*p* value
Sex							
Male	33 (80.5)	9 (19.5)	42 (17.3)	1			
Female	143 (71)	58 (29.9)	201 (82.7)	1.487 (0.670–3.302)	0.329		
Age in years							
≤20	69 (68.3)	32 (31.7)	101 (41.6)	1			
21–35	100 (77.5)	29 (22.5)	129 (53.1)	0.625 (0.347–1.127)	0.118	0.509 (0.216–1.201)	0.123
36–50	7 (53.8)	6 (46.2)	13 (5.3)	1.848 (0.575–5.944)	0.303	0.981 (0.179–5.378)	0.983
Educational level							
Illiterate	31 (60.8)	20 (29.2)	51 (21)	1			
Read and write	4 (75.0)	1 (25)	5 (2.1)	0.388 (0.040–3.722)	0.411	0.116 (0.009–1.488)	0.098
Primary: 1–4	13 (61.9)	9 (38.1)	22 (9)	1.073 (0.387–2.973)	0.892	2.119 (0.512–8.767)	0.300
Primary: 5–8	61 (74.4)	21 (25.6)	82 (34)	0.534 (0.252–1.129)	0.101	0.974 (0.335–2.834)	0.961
Secondary: 9–12	49 (76.5)	15 (23.5)	64 (26.3)	0.474 (0.212–1.063)	0.070	0.530 (0.169–1.662)	0.276
College and above	18 (94.3)	1 (5.7)	19 (7.8)	0.086 (0.011–0.697)	0.022	0.114 (0.007–1.906)	0.131
Service year							
<1 year	100 (75.2)	33 (24.8)	133 (54.7)	1			
≥1 year	76 (69.7)	34 (30.1)	110 (45.3)	1.356 (0.771–2.383)	0.291		
Training certified							
Yes	4 (80.0)	1 (20.0)	5 (2.1)	1			
No	172 (72.2)	66 (27.8)	238 (98)	1.535 (0.168–13.986)	0.704		
Fingernail status							
Trimmed	120 (85.7)	20 (14.3)	140 (57.6)	1			
Untrimmed	56 (54.4)	47 (45.6)	103 (42.3)	5.036 (2.731–9.285)	<0.001	4.067 (1.832–9.027)	0.001
Regular hand washing with detergent after toilet							
Yes	119 (83.2)	24 (16.8)	143 (59)	1			
No	57 (57.0)	43 (43.0)	100 (41)	3.740 (2.072–6.753)	<0.001	1.682 (0.709–3.987)	0.238
Preparing food during diarrhea, GI discomfort							
Yes	70 (74.5)	24 (25.5)	94 (38.6)	0.845 (0.472–1.515)	0.572		
No	106 (71.1)	43 (29.9)	149 (61.3)	1			
Hand washing with soap before food preparation							
Yes	103 (88.0)	14 (22.0)	117 (48)	1			
No	73 (57.9)	53 (42.1)	126 (52)	5.341 (2.758–10.344)	<0.001	1.693 (0.679–4.222)	0.259
Availability of clean toilet facility							
Yes	103 (79.8)	26 (20.2)	129 (53)	1			
No	73 (64.0)	41 (36)	149 (61)	2.225 (1.251–3.951)	0.006	1.313 (0.556–3.099)	0.534
Using common knife for meat and vegetables							
Yes	42 (75.0)	14 (25.0)	56 (23)	0.843 (0.426–1.669)	0.624		
No	134 (71.6)	53 (28.4)	187 (77)	1			
Regular hand washing with soap before eating							
Yes	100 (81.3)	23 (18.7)	123 (50.6)	1			
No	76 (63.3)	44 (36.6)	120 (49.4)	2.517 (1.401–4.522)	0.002	1.401 (0.581–3.377)	0.453
Washing fruit before eating							
Yes	113 (87.6)	16 (12.4)	129 (53)	1			
No	63 (55.3)	51 (44.7)	114 (47)	5.717 (3.013–10.849)	<0.001	4.428 (1.857–10.56)	0.001
Regular shoe wearing habit							
Yes	124 (80.5)	30 (19.5)	154 (63.4)	1			
No	52 (58.4)	37 (41.6)	89 (36.6)	2.941 (1.646–5.254)	<0.001	2.311 (1.020–5.23)	0.045
Wearing kitchen coat during food processing							
Yes	94 (71.2)	38 (28.8)	132 (54.3)	1			
No	82 (73.9)	29 (26.1)	111 (45.7)	0.875 (0.496–1.542)	0.644		
Cleaning utensils							
Yes	145 (85.8)	24 (14.2)	154	1			
No	31 (41.9)	43 (58.1)	89	8.380 (4.453–15.771)	<0.001	5.690 (2.382–13.59)	<0.001

*Note.* AOR = adjusted odds ratio, COR = crude odds ratio, CI = confidence interval.

**Table 2:** Bivariate analysis of risk factors associated with *Salmonella* and *Shigella* isolates in food handlers in Motta town, North West Ethiopia.

Characteristics	Noncarrier *N* (%)	Carrier *N* (%)	OR (95% CI)	*p* value
Sex				
Male	39(92.8)	3(7.2)	1	
Female	194(96.5)	7(3.5)	0.469(0.12–1.9)	0.384
Age in years				
≤20	98(97.0)	3(3.0)	0.37(0.03–3.8)	0.402
21–35	123(95.3)	6(4.7)	0.59(0.06–5.3)	0.633
36–50	12(92.3)	1(7.7)	1	
Educational level				
Illiterate	49(96.1)	2(3.7)	0.74(0.06–8.6)	0.806
Read and write	4(80.0)	1(20.0)	4.5(0.23–88)	0.322
Primary: 1–4	22(91.7)	2(8.3)	1.8(0.15–21)	0.643
Primary: 5–8	78(97.5)	2(2.5)	0.52(0.45-0.04-5.2)	0.524
Secondary: 9–12	62(96.9)	2(3.1)	0.58(0.05–6.7)	0.665
College and above	18(94.7)	1(5.3)	1	
Total year of service				
<1 year	128(96.2)	5(3.8)	1	
≥1 year	105(95.4)	5(4.6)	1.219(0.344–4.324)	0.759
Fingernail status				
Trimmed	138(98.6)	2(1.4)	1	
Untrimmed	95(92.2)	8(7.8)	5.811(1.207–27.967)	0.020
Regular hand washing with soap after toilet				
Yes	140(97.9)	3(2.1)	1	
No	93(93.0)	7(7.0)	3.513(0.886–13.93)	0.097
Preparing food during diarrhea, GI discomfort				
Yes	90(95.7)	4(4.3)	1	
No	143(96.0)	6(4.0)	0.944(0.259–3.438)	1
Hand washing with soap before food preparation				
Yes	115(98.3)	2(1.7)	1	
No	118(93.6)	8(6.7)	3.9(0.811–18.75)	0.105
Availability of clean toilet				
Yes	126(97.7)	3(2.3)	1	
No	107(93.8)	7(6.2)	2.8(0.69–10.89)	0.197
Cutting raw meat and vegetables by using the same knife				
Yes	54(94.7)	3(5.3)	1	
No	179(96.2)	7(3.7)	0.28(0.078–1.006)	0.054
Regular hand washing with soap before eating				
Yes	121(98.4)	2(1.6)	1	
No	112(93.3)	8(6.7)	4.32(0.9–20.78)	0.058
Washing of fruit before eating				
Yes	126(97.7)	3(2.3)	1	
No	107(93.8)	7(6.2)	2.75(0.693–10.89)	0.197
Wearing hair cover during food processing				
Yes	138(97.2)	4(2.8)	1	
No	95(94.0)	6(6.0)	2.2(0.59–7.93)	0.327
Wearing kitchen gown during food preparation				
Yes	130(98.5)	2(1.5)	1	
No	103(92.8)	8(7.2)	5.04(1.049–24.29)	0.047
Cleaning utensils				
Yes	164(97.0)	5(3.0)	1	
No	69(93.2)	5(6.8)	2.4(0.667–8.47)	0.178

*Note.* OR = odds ratio, CI = confidence interval.

**Table 3:** Antimicrobial susceptibility patterns of *Salmonella and Shigella* among food handlers in Motta town, North West Ethiopia.

Antimicrobial agents	Antimicrobial susceptibility pattern (*Shigella* = 4, *Salmonella* = 6)					
	Resistance *N* (%)		Intermediate *N* (%)		Susceptible *N* (%)	
	*Salmonella* (*n* = 6)	*Shigella* (*n* = 4)	*Salmonella* (*n* = 6)	*Shigella* (*n* = 4)	*Salmonella* (*n* = 6)	*Shigella* (*n* = 4)
Amoxicillin-Clavulanate	3(50)	3(75)	0(0)	0(0)	3(50)	1(25)
Chloramphenicol	0(0)	1(25)	0(0)	0(0)	6(100)	3(75)
Cefotaxime	1(16.7)	1(25)	0(0)	1(25)	5(83.3)	2(50)
Ampicillin	6(100)	4(100)	0(0)	0(0)	0(0)	0(0)
Ciprofloxacin	0(0)	0(0)	0(0)	0(0)	6(100)	4(100)
Trimethoprim-Sulfamethoxazole	1(16.7)	0(0)	0(0)	0(0)	5(83.3)	4(100)
Tetracycline	5(83.3)	4(100)	1(16.7)	0(0)	0(0)	0(0)

## Data Availability

The data used to support the findings of this study are available from the corresponding author upon request.

## Abbreviations

CDC: Centers for Disease Control and Prevention
CLSI: Clinical and Laboratory Standard Institute
FBD: Food-borne diseases
FMoH: Federal Ministry of Health
IP: Intestinal parasite
IPIs: Intestinal parasitic infections
MDR: Multi drug resistant
NTDs: Neglected tropical diseases
SOP: Standard operational procedure
SPSS: Statistical Package for Social Sciences
WHO: World Health Organization

## Additional Points

In this study, single stool samples were collected from each study participants rather than the triplicate samples recommended for detecting intestinal parasites that are passed in the faeces intermittently, and this might compromise the prevalence of intestinal parasites.
